# Analytical validation of capillary blood Krebs von den Lungen-6 measurement using a high- sensitivity chemiluminescent immunoassay in pediatric patients

**DOI:** 10.3389/fmed.2026.1894242

**Published:** 2026-09-03

**Authors:** Chengyuan Zhao, Lingyun Shi, Dan Cao, Xianwei Zhang, Tong Cao

**Affiliations:** 1 Department of Clinical Laboratory Medicine, Children’s Hospital of Nanjing Medical University, Nanjing, China; 2 Nanjing Poclight Biotechnology Company Limited, Nanjing, China

**Keywords:** chemiluminescence immunoassay, human, interstitial lung diseases, Krebs von den Lungen-6 protein, pediatric

## Abstract

**Objective:**

This study evaluates the analytical validity of capillary blood for Krebs von den Lungen-6 (KL-6) detection using a high-sensitivity chemiluminescent immunoassay (CLIA) on the C5000 analyser and assesses agreement between capillary plasma and venous plasma measurements, with emphasis on methodological feasibility rather than clinical diagnostic performance.

**Methods:**

Analytical performance was assessed in terms of repeatability, accuracy and method comparison. Precision was evaluated using pediatric venous plasma samples at three concentration levels, each tested in 20 replicates. Accuracy was verified using manufacturer standards and external quality controls. Comparative analysis involved 80 paired venous plasma and capillary blood samples, with hematocrit (HCT)-based correction applied to address dilutional effects. Agreement between venous and capillary plasma measurements was evaluated using Passing–Bablok regression, Pearson correlation and Bland–Altman analysis. Pediatric and adult KL-6 levels were also profiled to evaluate age-specific differences.

**Results:**

The C5000 analyzer demonstrated excellent repeatability, with coefficients of variation below 3% across all concentration levels. Accuracy assessment showed minimal bias relative to the standards and controls. Passing–Bablok regression indicated proportional bias before HCT correction, with a slope of 0.769 (95% CI, 0.550–0.945). After correction, the slope was 1.004 (95% CI, 0.882–1.162) and the intercept was −7.03 U/mL (95% CI, −26.97 to 9.76). The mean capillary-minus-venous bias decreased from −48.32 to −3.47 U/mL, and the Pearson correlation coefficient increased from 0.958 to 0.966. Pediatric KL-6 levels were significantly lower than adult levels (*p* < 0.001), underscoring the need for pediatric-specific exploratory reference estimates and future diagnostic validation.

**Conclusion:**

High-sensitivity CLIA on the C5000 analyzer supports the analytical feasibility of KL-6 measurement in pediatric capillary blood. HCT correction improved agreement with venous plasma measurements, although further validation is required before the two sample types can be considered interchangeable for individual clinical assessment and before formal reference intervals can be established.

## 1 Introduction

### 1.1 Biomarker detection in pediatric clinical practice

The accurate quantification of serum biomarkers is critical for disease diagnosis, therapeutic monitoring and prognostic evaluation. Krebs von den Lungen-6 (KL-6) is a mucin glycoprotein (approx. 200–500 kDa following glycosylation), predominantly expressed by type II alveolar epithelial cells, that has emerged as a pivotal diagnostic biomarker for interstitial lung diseases (ILDs) (1). Elevated serum KL-6 levels demonstrate strong clinical correlations with disease progression in idiopathic pulmonary fibrosis, hypersensitivity pneumonitis and collagen vascular disease–associated ILDs (2, 3). Although KL-6 monitoring has become standard practice in adult ILD management (4), its clinical utility in pediatric populations remains incompletely established, with limited pediatric-specific data and validated cut-offs.

### 1.2 Challenges in pediatric biomarker sampling

Conventional venous blood collection faces inherent limitations in children, particularly those requiring serial monitoring for chronic pulmonary conditions (5). Anatomical constraints (reduced vascular caliber) combined with psychological distress during phlebotomy frequently lead to procedural complications and caregiver anxiety. Furthermore, repeated venipuncture imposes substantial burdens on healthcare resources. These barriers underscore the urgent need for child-friendly sampling methodologies.

### 1.3 Capillary blood as a promising alternative

Minimally invasive capillary blood sampling has emerged as a feasible approach for pediatric patients, providing advantages such as procedural simplicity, reduced pain and improved compliance (6, 7). Since KL-6 is a plasma protein, capillary blood is centrifuged to obtain plasma prior to analysis to ensure physiologically meaningful measurements. Nevertheless, clinical implementation is limited by pre-analysis factors, including small sample volume and potential haemodilution effects, necessitating careful validation for accurate biomarker quantification (8).

### 1.4 Technological advancements in chemiluminescence detection

The recent development of the C5000 chemiluminescence immunoanalyser (Nanjing Poclight Biotechnology Co., Ltd, China) offers innovative solutions through a heterogeneous chemiluminescent immunoassay (CLIA) platform. This system employs magnetic microparticles as solid-phase carriers and adenosine ester–labeled antibodies for high-sensitivity KL-6 detection. Magnetic separation and wash steps remove unbound components, enabling accurate measurement of plasma or capillary-derived samples within 5–10 min (9). Crucially, the adenosine ester–based detection demonstrates superior resistance to hemoglobin interference compared with conventional fluorescent markers (e.g., Cy5), as evidenced by successful KL-6 quantification in adult venous blood (10).

### 1.5 Rationale and study objectives

Despite proven utility in adults, the performance characteristics of C5000-based KL-6 detection remain unestablished in pediatric populations. This study systematically evaluates the analytical validity of the C5000 platform for KL-6 quantification and the agreement between capillary plasma and venous plasma measurements. Through precision assessment, calibration with certified standards and paired-sample comparisons, we aim to provide preliminary evidence supporting the methodological feasibility of using capillary blood as a minimally invasive alternative to venous blood. Clinical diagnostic performance in pediatric ILD will require further validation in well-characterized cohorts.

## 2 Methods

### 2.1 Study participants

This single-center observational study evaluated the feasibility of using capillary blood for KL-6 measurement in pediatric populations. Capillary blood samples were collected from cohort 1, comprising 276 adults (146 men, 130 women) and 110 healthy children (71 boys, 39 girls), and cohort 2, comprising 37 pediatric patients with severe pneumonia and 41 with mild pneumonia. Before enrolment, adult participants underwent medical history review, physical examination and necessary laboratory screening. Individuals were excluded if they had a history or current evidence of chronic respiratory disease, including interstitial lung disease, pulmonary fibrosis, chronic obstructive pulmonary disease, asthma, bronchiectasis or pulmonary tuberculosis; connective tissue disease or other autoimmune/inflammatory disorders known to affect KL-6 levels; acute infection or fever; malignancy; active hepatic or renal dysfunction; hematological disease; organ transplantation; recent surgery, blood transfusion, immunosuppressive therapy or antitumor treatment; pregnancy or lactation; and current smoking or a heavy smoking history. Individuals with damaged forearm skin, local skin disease or other conditions unsuitable for blood collection were also excluded.

Ethical approval was granted by the Ethics Committee of the Children’s Hospital of Nanjing Medical University. Written informed consent was obtained from all adult participants and from the parents or legal guardians of all children. All samples were anonymised prior to analysis, and data were handled in accordance with the Declaration of Helsinki and local data protection regulations.

### 2.2 Analytical performance validation of C5000 analyzer

**FIGURE 1:**
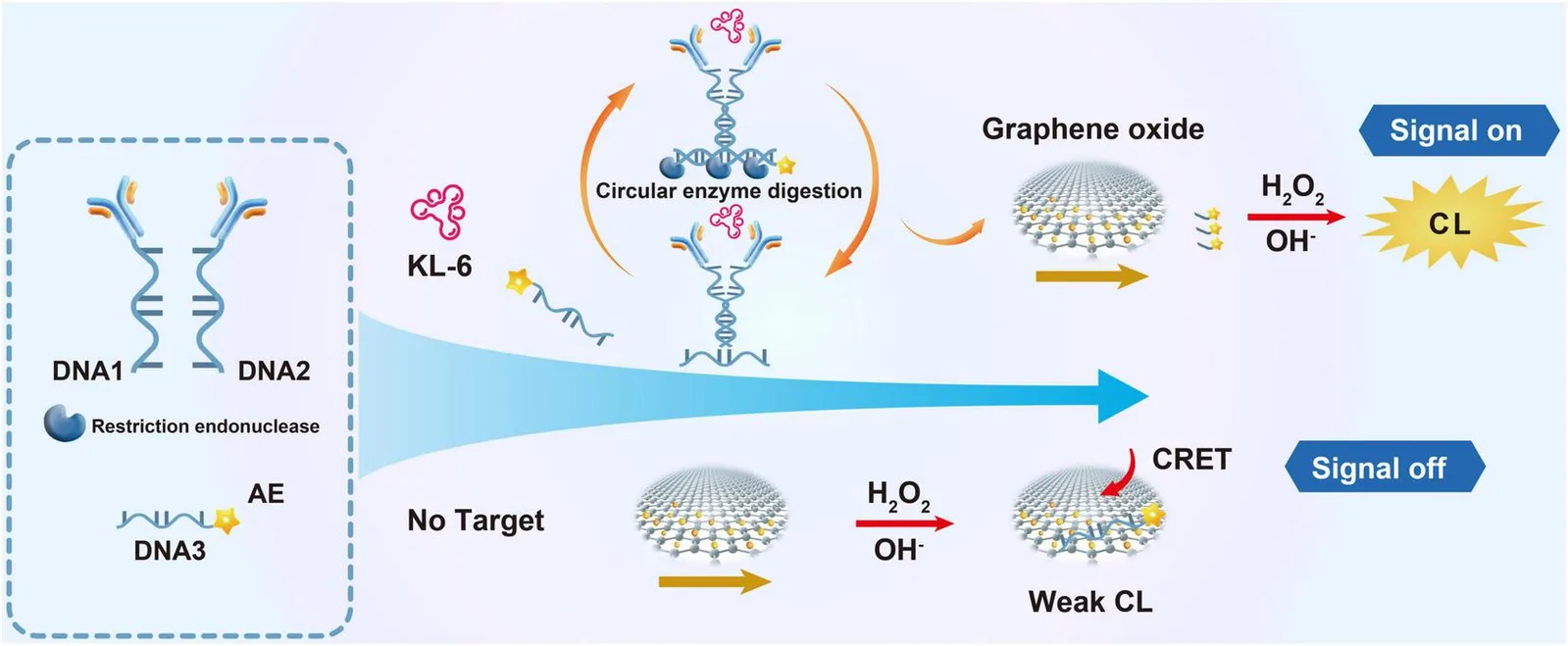
Basic Principle of C5000 Instrument for Detecting KL-6 Biomarker. The detection solution used by the analyser includes DNA1-antibody1 conjugates, DNA2-antibody2 conjugates, DNA3 labeled with acridinium ester (AE), and antioxidant (AOD) bound to graphene oxide (GO). DNA3 has eight bases that can form complementary pairs with DNA1 and DNA2. When KL-6 (the target protein) is present in the sample, the antibodies on the two antibody-DNA complexes form a double-antibody sandwich structure, bringing DNA1 and DNA2 close enough to form a proximal complex and hybridize with DNA3. This complex is minimally adsorbed by GO, and the AOD conjugated to GO has little effect on the luminescence of AE on the complex. In the absence of KL-6 protein in the sample, DNA3 is adsorbed onto the surface of GO through π–π stacking interactions. The AE labeled at the end of DNA3 cannot oxidize to luminesce due to the presence of the antioxidant on GO. Even minimal chemiluminescence is quenched by chemical luminescence resonance energy transfer (CRET).

The C5000 platform measures KL-6 using a sandwich CLIA based on specific antigen–antibody interactions (Figure 1). In this assay, KL-6 in plasma binds to capture antibodies immobilized on magnetic microparticles, forming a stable complex. A secondary antibody conjugated with a chemiluminescent label binds to the antigen, generating light emission proportional to KL-6 concentration. Unbound components are removed through magnetic separation and wash steps, minimizing background signal and ensuring high specificity and sensitivity. The platform was originally validated for venous plasma samples. In this study, a hematocrit (HCT)-based correction algorithm was applied to evaluate its performance on capillary blood–derived plasma while noting that the correction model is proprietary and its detailed parameters are not fully disclosed.

#### 2.2.1 Precision assessment

Following China National Accreditation Service for Conformity Assessment-GL037:2019 guidelines (11), intra-assay precision was assessed using pediatric venous blood samples grouped into three KL-6 concentration ranges: high (1,300–1,500 U/mL), medium (300–400 U/mL) and low (100–200 U/mL). Each group underwent 20 consecutive measurements under identical operational conditions. Precision was quantified by calculating the coefficient of variation (CV), with acceptability criteria set at CV < 3%.

#### 2.2.2 Accuracy verification

Accuracy was determined using certified reference materials (CRM) provided by the manufacturer (195 and 910 U/mL) and external quality controls (QCs) from Fuji Rebio (Tokyo, Japan) and Hunan Yonghe Yangguang Biotech (Hunan, China). Two replicates per concentration were analysed daily over 5 consecutive days. Method comparability was assessed through linear regression analysis against established reference methods – commercially available kits from Fuji Rebio and Hunan Yonghe Yangguang Biotech.

### 2.3 Methodological comparison: venous plasma versus capillary plasma

Paired venous plasma and capillary blood samples (*n* = 80) were collected from healthy children and pediatric patients with pneumonia. Capillary blood was centrifuged to obtain plasma prior to analysis, ensuring comparability with venous plasma. To address potential HCT-induced matrix effects, a proprietary correction algorithm that incorporates HCT and reagent lot-specific parameters was applied. The algorithm was derived from a predictive model trained on a dataset of 1,200 samples; details regarding the model type and validation are proprietary to the manufacturer. Capillary blood was collected by uniformly trained nursing staff according to the institutional standard operating procedure for peripheral capillary blood sampling. Participants were placed in a seated or semi-recumbent position. The ulnar side of the fingertip of the left ring finger was selected as the puncture site, provided that the local skin was intact and free of infection. Following disinfection with 75% ethanol and complete air drying, a single-use safety lancet was used for puncture. The first drop of blood was discarded. Blood flow was promoted by gentle and intermittent pressure below the puncture site, with excessive squeezing, repeated massage or vigorous manipulation avoided to reduce tissue-fluid contamination and hemolysis. Blood was then collected into a microcollection tube, mixed immediately according to reagent requirements and centrifuged within the specified time window to obtain capillary plasma. Following sampling, sterile cotton was applied with pressure to stop bleeding, and the puncture site was observed for local reactions. To reduce bias, nursing staff were responsible only for sample collection and coding and did not participate in assay operation or result interpretation. Laboratory personnel received coded samples only and were blinded to sample source and clinical grouping; statistical analysis was performed following data locking and subsequent unblinding.

### 2.4 Statistical analysis

All analyses were performed using R version 4.2.2 (R Foundation for Statistical Computing, Vienna, Austria). Continuous variables were summarized as mean ± SD or median (IQR), as appropriate. Normality was assessed using the Shapiro–Wilk test. Intra-assay precision was reported as CV. Accuracy was evaluated by comparing measured KL-6 concentrations with expected values of CRMs and external QCs using paired t-tests. Method comparison with established reference assays (Fuji Rebio, Hunan Yonghe) was assessed via linear regression, Pearson correlation and Bland–Altman analysis.

Agreement between venous and capillary plasma KL-6 measurements before and after HCT correction was evaluated using Passing–Bablok regression, Pearson correlation and Bland–Altman analysis. Venous plasma was treated as the reference measurement and capillary plasma as the comparison measurement. Passing–Bablok intercepts and slopes were reported with 95% confidence intervals; constant bias was considered present when the confidence interval for the intercept excluded 0, and proportional bias was considered present when the confidence interval for the slope excluded 1. Bland–Altman bias was calculated as the capillary measurement minus the venous measurement, together with the corresponding 95% limits of agreement. Paired differences between capillary and venous plasma measurements were assessed using the Wilcoxon signed-rank test.

Values reported as <50 U/mL were treated as left-censored at the lower reportable limit. The primary method-comparison analysis excluded paired observations containing at least one left-censored result and therefore included 76 fully quantifiable pairs. A sensitivity analysis included all 80 pairs by replacing values reported as <50 U/mL with 49.9 U/mL. Passing–Bablok regression was selected instead of Deming regression because separate estimates of analytical imprecision for the venous and capillary sample matrices were unavailable to specify the error-variance ratio required for Deming regression. To evaluate the performance of HCT correction across the observed HCT range, residual bias was defined as HCT-corrected capillary KL-6 minus venous plasma KL-6. Associations of HCT with residual bias and absolute residual error were assessed using Spearman correlation. Corrected measurement differences were also summarized across observed HCT strata of ≤ 35.0%, 35.1%–40.0% and >40.0%, with between-stratum comparisons performed using the Kruskal–Wallis test.

An exploratory threshold-classification analysis was performed at a KL-6 concentration of 500 U/mL using venous plasma classification as the analytical reference. Analytical sensitivity, specificity, false-positive and false-negative classifications, and exact 95% confidence intervals were calculated for HCT-corrected capillary measurements. The number of venous plasma measurements within 400–600 U/mL was also recorded to characterize observations near the threshold. Pediatric-specific KL-6 reference estimates were explored by grouping data according to age, sex and disease severity. Differences between two groups were assessed using Student’s *t*-test, whereas comparisons among multiple groups were evaluated using one-way analysis of variance. Statistical significance was defined as *p* < 0.05. Reporting was considered against Standards for Reporting of Diagnostic Accuracy Studies recommendations where applicable.

## 3 Results

### 3.1 Analytical validation outcomes

**FIGURE 2:**
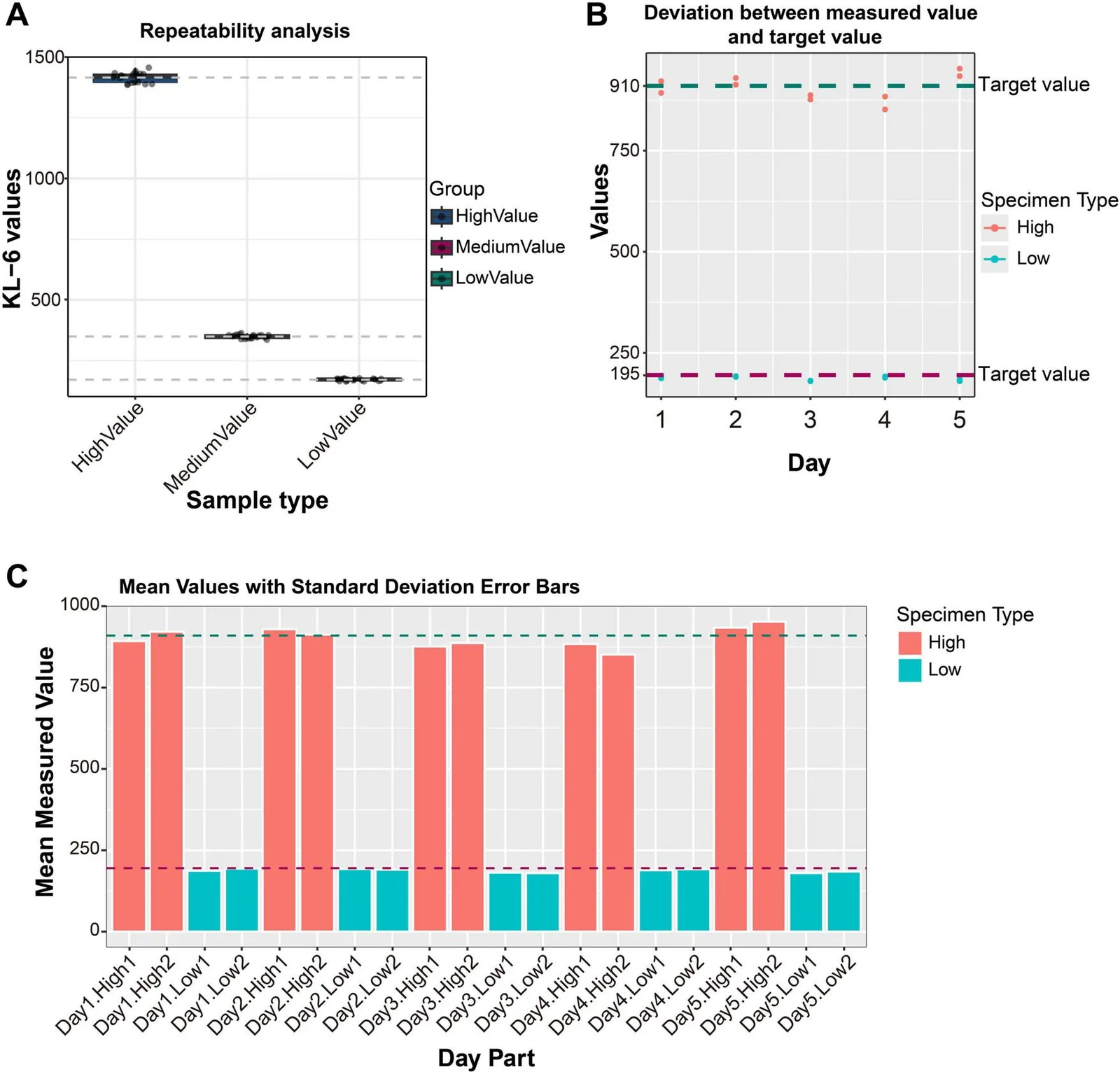
Reliability and stability of the C5000 analyser. **(A)** The coefficients of variation (CV) for high, medium, and low values are within a controllable range. **(B, C)** Accuracy validation results indicate that the differences between the detected values and the target values are not significant.

Regarding precision, intra-assay CVs were 1.49% (high), 2.18% (medium) and 2.62% (low), meeting predefined criteria (Table 1, Figure 2).

**TABLE 1:** Precision study results for high, medium, and low values.

Group	Mean	SD	CV
HighValue	1415.641	21.04418	0.014865
MediumValue	348.7705	7.59164	0.021767
LowValue	170.8605	4.483533	0.026241

**FIGURE 3:**
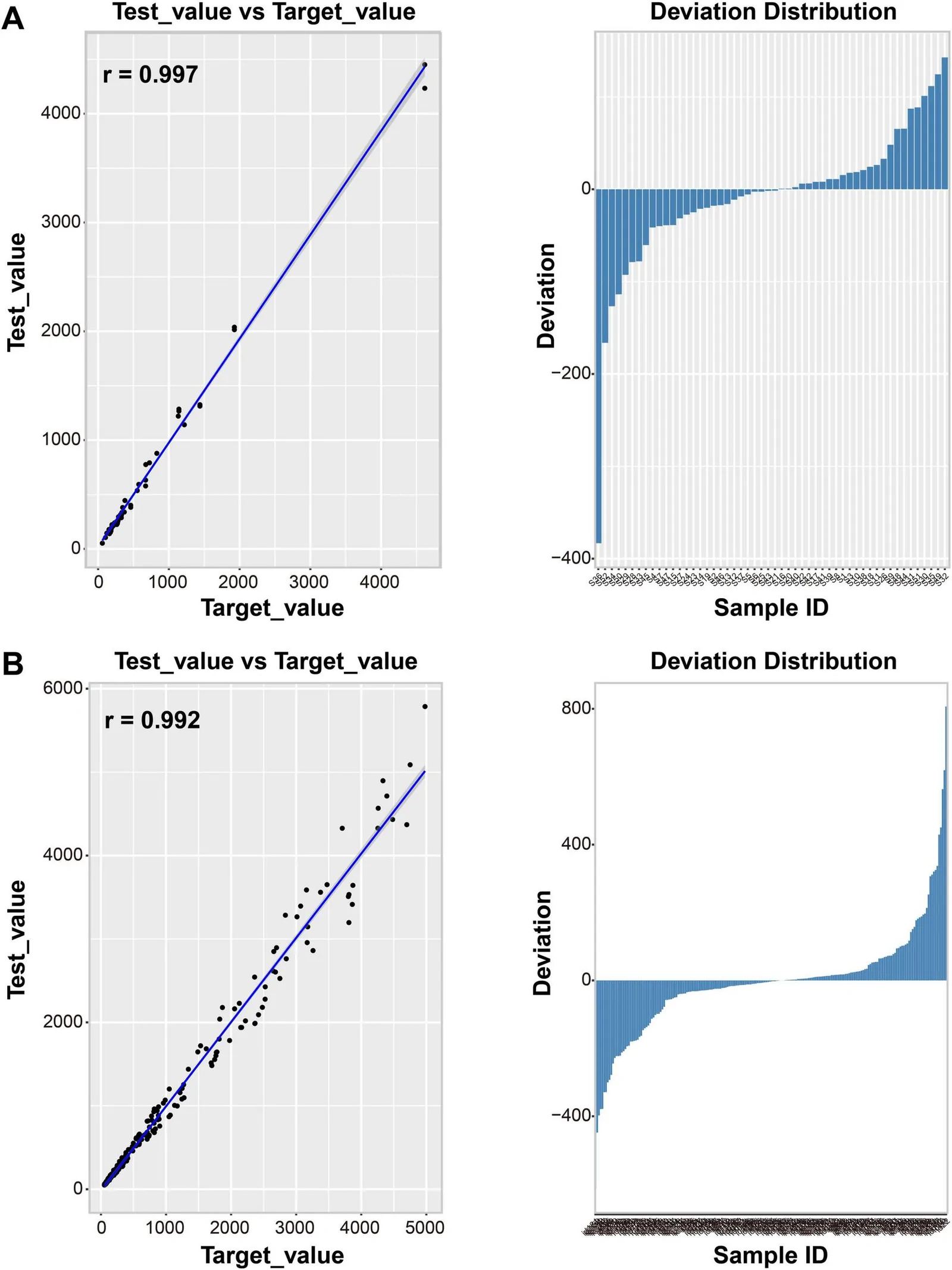
Comparative analysis of target and detected values using standards from two companies. **(A)** Analysis results of standards provided by Fujirebio Inc., Japan, indicate close agreement between target and detected values, with no significant difference and strong correlation (*p* < 0.001 for correlation). **(B)** Analysis results of standards provided by Hunan Yonghe Yangguang Biotechnology Co., Ltd., indicate close agreement between target and detected values, with no significant difference and strong correlation (*p* < 0.001 for correlation).

For accuracy, no significant bias was observed between measured and expected values across all CRMs (*p* > 0.96, paired *t*-test). A detailed comparison between detected and target values is summarized in Table 2. Method comparison demonstrated excellent correlation with reference methods (Fuji Rebio: *r* = 0.997, *p* < 0.001; Hunan Yonghe: *r* = 0.992, *p* < 0.001) (Figure 3).

**TABLE 2:** Accuracy validation of detected values versus target values.

Target value (U/mL)	Type	Day_Part	Mean	SD	N	Deviation
910	High	Day1.High1	892.8	NA	1	−17.2
	High	Day1.High2	921.61	NA	1	11.61
	High	Day2.High1	929.55	NA	1	19.55
	High	Day2.High2	912.97	NA	1	2.97
	High	Day3.High1	876.37	NA	1	−33.63
	High	Day3.High2	887.09	NA	1	−22.91
	High	Day4.High1	883.78	NA	1	−26.22
	High	Day4.High2	851.72	NA	1	−58.28
	High	Day5.High1	934.17	NA	1	24.17
	High	Day5.High2	952.7	NA	1	42.7
195	Low	Day1.Low1	187.07	NA	1	−7.93
	Low	Day1.Low2	193.79	NA	1	−1.21
	Low	Day2.Low1	192.55	NA	1	−2.45
	Low	Day2.Low2	190.47	NA	1	−4.53
	Low	Day3.Low1	181.45	NA	1	−13.55
	Low	Day3.Low2	179.82	NA	1	−15.18
	Low	Day4.Low1	188.72	NA	1	−6.28
	Low	Day4.Low2	191.78	NA	1	−3.22
	Low	Day5.Low1	180.02	NA	1	−14.98
	Low	Day5.Low2	184.7	NA	1	−10.3

### 3.2 Agreement between venous and capillary plasma following haematocrit correction

**FIGURE 4:**
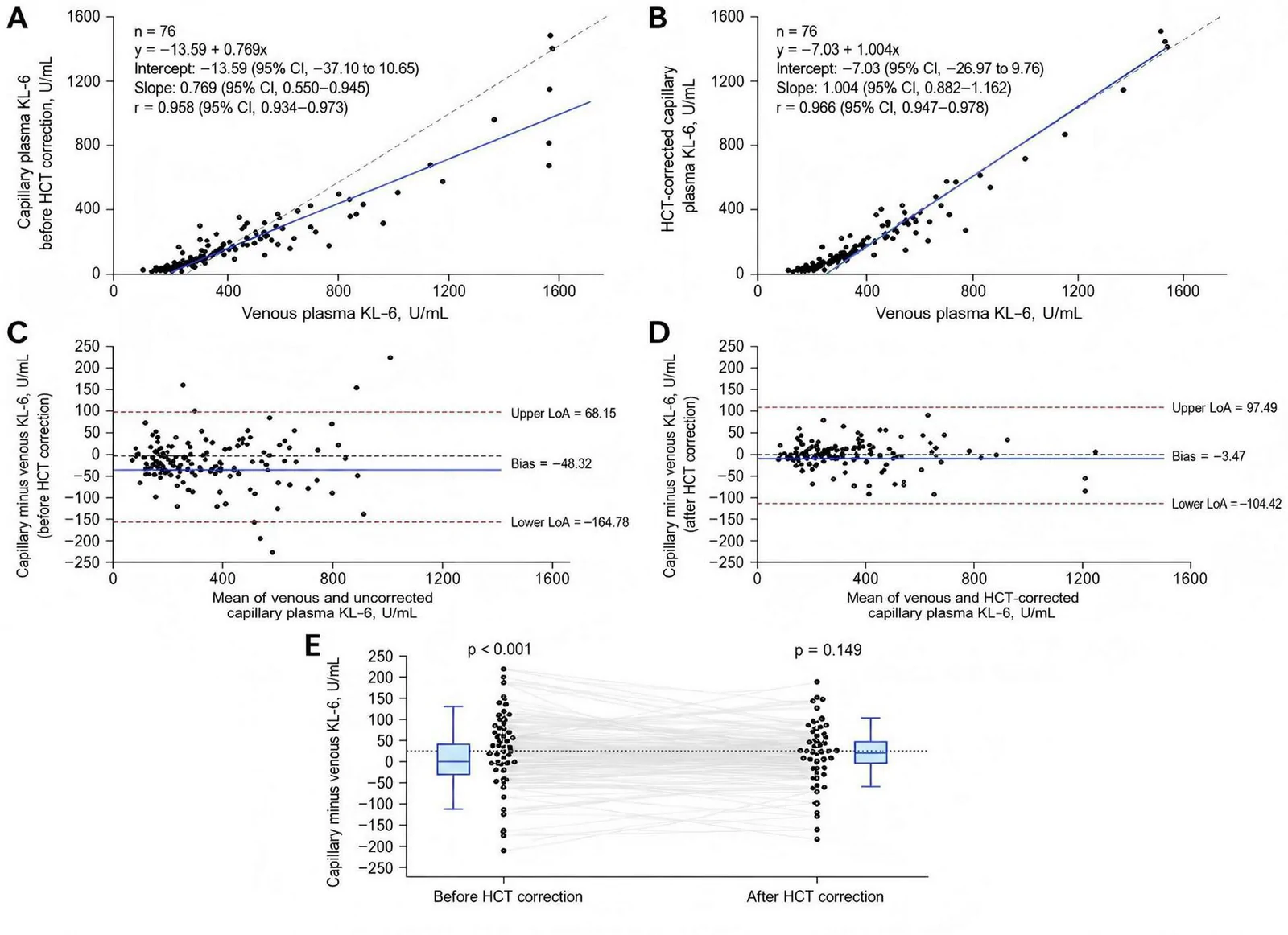
Agreement between venous and capillary plasma KL-6 measurements before and after hematocrit correction. **(A, B)** Passing–Bablok regression before and after HCT correction, respectively. Solid blue lines indicate the regression fits, and grey dashed lines indicate the identity line. **(C, D)** Bland–Altman analyses before and after HCT correction, respectively. Solid blue lines indicate mean bias, red dashed lines indicate the 95% limits of agreement, and black dashed lines indicate zero difference. **(E)** Paired capillary-minus-venous KL-6 differences before and after HCT correction; grey lines connect measurements from the same participant. *P* values were obtained using two-sided Wilcoxon signed-rank tests. The primary analysis included 76 fully quantifiable pairs; sensitivity analyses including all 80 pairs are shown in Supplementary Table 2. CI, confidence interval; HCT, hematocrit; KL-6, Krebs von den Lungen-6; LoA, limits of agreement.

Four of the 80 paired observations contained at least one KL-6 result reported as <50 U/mL; therefore, the primary method-comparison analysis included 76 fully quantifiable pairs (Supplementary Tables 1 and 2). Before HCT correction, Passing–Bablok regression yielded an intercept of −13.59 U/mL (95% CI, −37.10 to 10.65) and a slope of 0.769 (95% CI, 0.550–0.945). Because the confidence interval for the slope excluded 1, proportional bias was present between uncorrected capillary and venous plasma measurements. The Pearson correlation coefficient was 0.958 (95% CI, 0.934–0.973) (Figure 4A; Supplementary Table 2).

After HCT correction, the Passing–Bablok equation was: corrected capillary KL-6 = −7.03 + 1.004 × venous plasma KL-6. The intercept was −7.03 U/mL (95% CI, −26.97 to 9.76), and the slope was 1.004 (95% CI, 0.882–1.162). The confidence intervals included 0 and 1, respectively, indicating no statistically detectable constant or proportional bias after correction. The Pearson correlation coefficient was 0.966 (95% CI, 0.947–0.978) (Figure 4B; Supplementary Table 2).

Bland–Altman analysis showed that the mean capillary-minus-venous bias decreased from −48.32 U/mL before correction to −3.47 U/mL after correction. The corresponding 95% limits of agreement were −164.78 to 68.15 U/mL before correction and −104.42 to 97.49 U/mL after correction (Figures 4C, D; Supplementary Table 2). The paired difference was significant before correction (*p* < 0.001) but not after correction (*p* = 0.149) (Figure 4E; Supplementary Table 2). Sensitivity analyses including all 80 pairs produced materially similar Passing–Bablok results before and after correction (Supplementary Table 2).

Across the 76 fully quantifiable pairs, HCT ranged from 18.7% to 44.1%. HCT was not significantly correlated with the corrected capillary-minus-venous difference (Spearman ρ = 0.115, *p* = 0.324) or with the absolute corrected difference (ρ = −0.017, *p* = 0.887). Mean corrected biases were −12.19, −6.08 and 12.05 U/mL for HCT strata of ≤35.0%, 35.1%–40.0% and >40.0%, respectively, with no significant between-stratum difference (*p* = 0.750) (Supplementary Table 3). At the exploratory 500 U/mL threshold, HCT-corrected capillary measurements reproduced the venous classification in all 76 pairs, including 2 of 2 measurements ≥ 500 U/mL and 74 of 74 measurements < 500 U/mL, corresponding to an analytical sensitivity of 100% (95% CI, 15.8%–100%) and specificity of 100% (95% CI, 95.1%–100%). No venous plasma measurements were within 400–600 U/mL.

### 3.3 Exploratory pediatric Krebs von den Lungen-6 reference estimates

**FIGURE 5:**
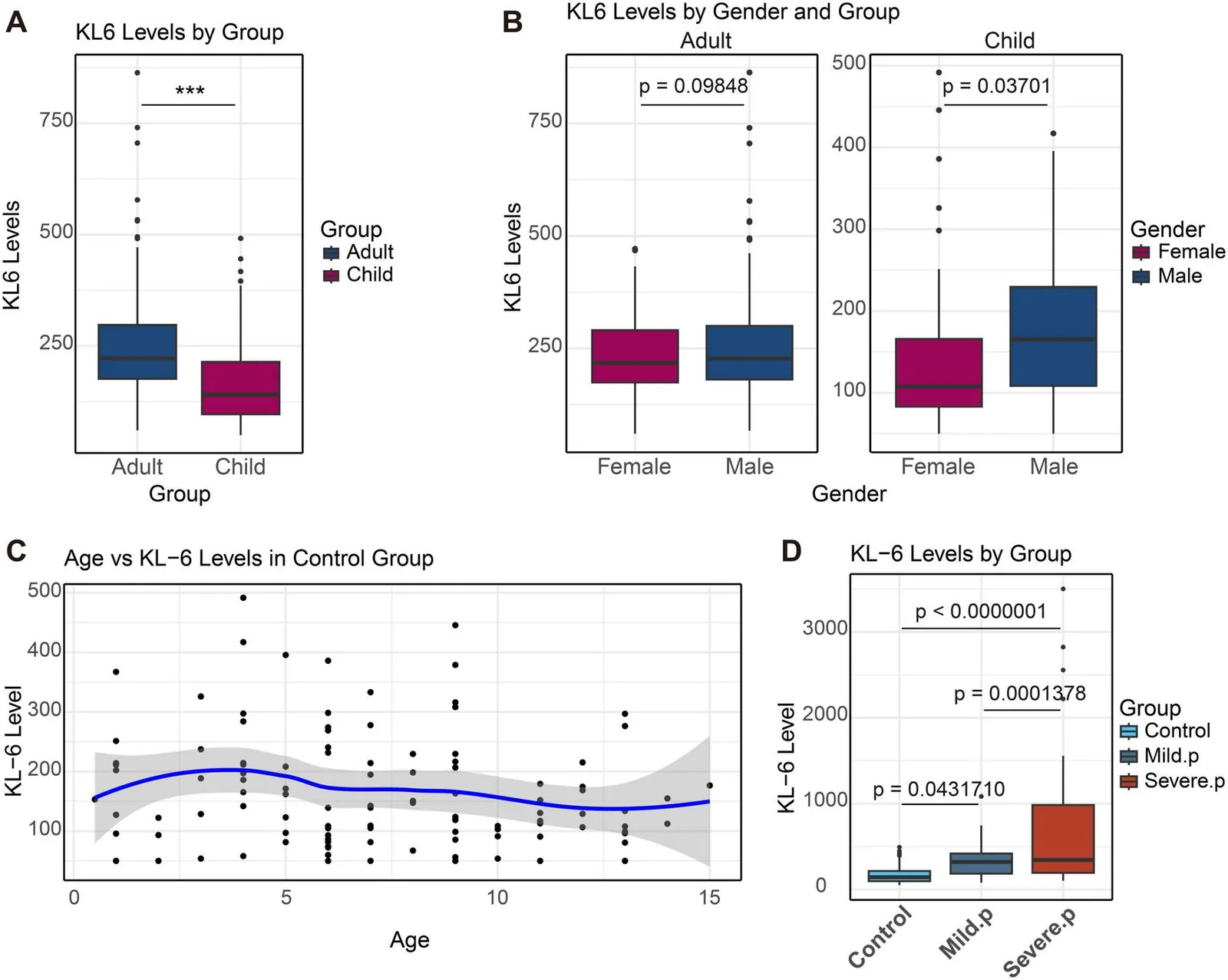
Exploratory KL-6 levels in pediatric vs. adult populations and by pneumonia severity. **(A)** The mean peripheral blood KL-6 level in the pediatric population is significantly lower than that in adults (*p* < 0.001). **(B)** There is no statistically significant difference in KL-6 levels between adult males and females. **(C)** In healthy children, KL-6 levels do not exhibit significant fluctuations with increasing age. **(D)** Under pathological conditions, KL-6 expression levels show a positive correlation with the severity of pneumonia; levels in severe pneumonia are significantly higher than those in mild pneumonia, and levels in mild pneumonia are significantly higher than those in healthy controls. These values should be interpreted as exploratory rather than formal reference intervals.

All reference estimates in this section are derived from capillary plasma samples. These values should be interpreted as exploratory rather than as formal reference intervals. Exploratory reference estimates include the following: (i) age dependency: pediatric levels (mean ± SD: 128 ± 34 U/mL) were significantly lower than those of adults (289 ± 67 U/mL, *p* = 5.28e-10), (ii) sexual dimorphism: significant gender differences were observed in children (boys: 142 ± 38 vs. girls: 109 ± 29 U/mL, *p* = 0.037) but absent in adults (*p* = 0.098) and (iii) disease correlation: KL-6 elevation paralleled pneumonia severity (severe: 415 ± 121 > mild: 267 ± 89 > controls: 128 ± 34 U/mL, *p* < 0.001). See Figure 5 for details.

## 4 Discussion

Although our study used pneumonia cohorts as a model of pulmonary epithelial injury to evaluate assay dynamic range and responsiveness, the implications for pediatric ILD management remain methodological rather than clinical. Childhood ILDs differ aetiologically from adult counterparts, with surfactant dysfunction mutations (e.g., *SFTPB*, *SFTPC*) accounting for >30% of cases in children under 2 years of age (12). Elevated KL-6 levels in these genetic disorders may reflect compensatory type II pneumocyte hyperplasia rather than pure fibrotic activity, underscoring the need for age-stratified interpretation. Notably, neonatal KL-6 levels in *ABCA3* mutation–associated ILD reach 2,800 U/mL (13), suggesting biomarker dynamics that are distinct from adult idiopathic pulmonary fibrosis. This biological heterogeneity supports the need for pediatric validation before adult thresholds (≥500 U/mL) can be applied in children (14). This study therefore provides analytical groundwork for capillary KL-6 measurement but does not establish clinical diagnostic performance in pediatric ILD.

This study established the analytical validity of the C5000 chemiluminescence platform for KL-6 quantification in pediatric peripheral blood, addressing two gaps: the need for a minimally invasive sampling approach and limited age-specific reference data. Our data confirmed that HCT-related effects significantly influenced uncorrected capillary plasma KL-6 measurements. Passing–Bablok regression identified proportional bias before correction, whereas the confidence intervals for the slope and intercept included 1 and 0, respectively, after correction. The proprietary correction model substantially reduced the mean bias, but the model parameters are not fully disclosed and therefore require independent validation. Careful interpretation remains important because KL-6 concentrations may also be influenced by clinical and laboratory confounders in connective tissue disease populations (15). The present findings support measurement of KL-6 from capillary blood-derived plasma obtained by centrifuging capillary samples, but they do not by themselves establish diagnostic cut-offs, longitudinal clinical utility or unrestricted interchangeability with venous plasma. The remaining Bland–Altman limits of agreement indicate residual individual-level variability, which should be addressed in future prospective validation studies.

The precision of the C5000 analyser (CV < 3%) meets rigorous standards for serological monitoring (16). Its assay design allows KL-6 measurement from capillary blood–derived plasma with minimal sample handling, which is advantageous because delayed processing can increase KL-6 degradation by 8.2% per hour in unprocessed blood (17). The 5–10-min turnaround time may be useful in pediatric workflows, although the present study did not evaluate point-of-care diagnostic impact or patient outcome benefit. Compared with conventional enzyme-linked immunosorbent assay protocols requiring 3–4 h (18), the C5000 platform is operationally more suitable for small-volume pediatric sampling. Moreover, the system achieves a limit of detection of 15 U/mL, supporting analytical sensitivity, but clinical threshold performance still requires dedicated validation (19).

The markedly lower KL-6 levels in children could reflect maturational changes in type II pneumocyte activity. The observed sexual dimorphism in our pediatric cohort (boys > girls) invites the formulation of explanatory hypotheses and may involve hormonal influences, but this requires further investigation. The KL-6 gradient across pneumonia severity groups mirrors its established role in adult acute lung injury (13), suggesting conserved biomarker functionality.

Although pioneering in pediatric KL-6 assay development, our study has the following limitations: (i) single-center design with restricted ethnic diversity (Chinese Han population), limiting the applicability of our findings to other racial or ethnic groups; (ii) absence of longitudinal data and adjudicated clinical outcomes; (iii) limited validation in a representative pediatric fibrotic ILD cohort; (iv) the HCT-based correction algorithm used in this study was provided by the manufacturer without full disclosure of model parameters or independent validation; and (v) although no significant HCT-dependent residual bias was detected across the observed HCT range of 18.7%–44.1%, truly extreme HCT values were sparsely represented, and performance outside this range remains uncertain. In addition, although HCT-corrected capillary measurements reproduced venous classification at the exploratory 500 U/mL threshold, only two venous measurements exceeded this threshold and none were within 400–600 U/mL, limiting assessment of potential misclassification near the clinical cut-off. The performance of capillary sampling in patients with connective tissue disease–associated microangiopathy, such as systemic sclerosis with digital ulcers, also remains uncertain. Raynaud phenomenon, impaired digital perfusion, fingertip scarring and active digital ulcers may make fingertip blood collection difficult and increase susceptibility to preanalytical error from repeated pressure or manipulation; dedicated paired venous–capillary validation is therefore required before this sampling approach is applied in systemic sclerosis–associated ILD. Laboratories seeking to implement this approach should verify the algorithm’s performance locally. Further work should establish multicenter pediatric reference intervals (*n* ≥ 1,000) using Clinical Laboratory Standards Institute EP28-A3c guidelines (20) and formally validate diagnostic performance near clinically relevant cut-offs.

In conclusion, the C5000 chemiluminescence platform supports KL-6 measurement in pediatric capillary plasma and demonstrates methodological feasibility as a less invasive sampling approach. HCT correction substantially improved agreement with venous plasma measurements, although residual individual-level variability remains and further validation is required before the two sample types can be considered interchangeable. These findings provide a basis for early detection and disease monitoring studies, but further multicentre work is needed to establish pediatric reference intervals, validate diagnostic performance and test rare ILD subtypes.

## Data Availability

The original contributions presented in the study are included in the article/Supplementary material, further inquiries can be directed to the corresponding authors.
