# Supplementary material for: Analytical validation of capillary blood Krebs von den Lungen-6 measurement using a high- sensitivity chemiluminescent immunoassay in pediatric patients
Source: Front Med (Lausanne). 2026 Sep 3;13:1894242. doi: 10.3389/fmed.2026.1894242 (PMC13581673; doi:10.3389/fmed.2026.1894242)
Supplement: Supplementary file 1 [file Table_1.docx]

**Supplementary Table 1.** Participant-level paired KL-6 concentrations in venous plasma and capillary plasma before and after haematocrit correction

| Samples | Venous plasma KL-6, U/mL | Capillary plasma KL-6 before HCT correction, U/mL | Capillary plasma KL-6 after HCT correction, U/mL | HCT, % |
| --- | --- | --- | --- | --- |
| S1 | 239.58 | 201.77 | 233.84 | 41.6 |
| S2 | 154.36 | 125.97 | 209.97 | 34.3 |
| S3 | 240.93 | 172.76 | 222.8 | 39.5 |
| S4 | 254.23 | 196.06 | 231.11 | 35.2 |
| S5 | 303.56 | 250.72 | 266.65 | 31.3 |
| S6 | 268.64 | 223 | 246.39 | 37.6 |
| S7 | 1001.39 | 970.23 | 970.15 | 35.2 |
| S8 | 147.57 | 113.99 | 204.32 | 42.3 |
| S9 | 120.41 | 99.29 | 193.09 | 18.7 |
| S10 | 196.25 | 126.3 | 210.1 | 39.6 |
| S11 | 150.74 | 92.31 | 184.75 | 37.1 |
| S12 | 178.18 | 133.3 | 212.5 | 44.1 |
| S13 | 265.27 | 247.66 | 264.27 | 38.4 |
| S14 | 183.21 | 94.46 | 187.6 | 40.4 |
| S15 | 221.54 | 204.17 | 235.08 | 38.5 |
| S16 | 363.86 | 386.78 | 387.54 | 38.3 |
| S17 | 243.13 | 318.02 | 324.12 | 39.7 |
| S18 | 131.57 | 166.45 | 221.1 | 36.8 |
| S19 | 288.97 | 156.29 | 218.61 | 37.8 |
| S20 | 66.88 | 59.69 | 82.32 | 36 |
| S21 | 1285.21 | 1496.94 | 1498.17 | 42.4 |
| S22 | 166.67 | 90.05 | 170.22 | 31.3 |
| S23 | 250.55 | 146.14 | 230.29 | 40.3 |
| S24 | 74.16 | 65.9 | 101.66 | 35.6 |
| S25 | 117.67 | 60.87 | 86.86 | 33.2 |
| S26 | 59.86 | 55.31 | 71.21 | 40.8 |
| S27 | 85.02 | 61.36 | 88.27 | 41.4 |
| S28 | 164.45 | 147.18 | 223.84 | 39.9 |
| S29 | 77.98 | 59.97 | 84.27 | 29.8 |
| S30 | 99.4 | 53.31 | 65.7 | 40.2 |
| S31 | 100.4 | 63.14 | 93.44 | 38.8 |
| S32 | 131.81 | 57.8 | 78.1 | 39.3 |
| S33 | 67.6 | 56.46 | 74.39 | 36.2 |
| S34 | 132.99 | 64.72 | 98.1 | 40.5 |
| S35 | 89.34 | 54.93 | 70.16 | 39.5 |
| S36 | 305.5 | 116.7 | 209.42 | 38 |
| S37 | 63.07 | <50 | <50 | 42.6 |
| S38 | 121.03 | 71.08 | 117.78 | 37.5 |
| S39 | 80.14 | 58.43 | 79.91 | 34.9 |
| S40 | 188.57 | 103.25 | 193.64 | 41.9 |
| S41 | <50 | 55.63 | 72.08 | 34.1 |
| S42 | 131.16 | 60.62 | 86.14 | 36.1 |
| S43 | 149.27 | 75.03 | 130.12 | 39 |
| S44 | 216.5 | 96.7 | 183.24 | 36.4 |
| S45 | 93.44 | 58.51 | 80.15 | 38.3 |
| S46 | 152.38 | 62.23 | 90.76 | 40.8 |
| S47 | 81.74 | 55.31 | 71.19 | 38.1 |
| S48 | 98.78 | 62.13 | 90.49 | 38.9 |
| S49 | 111.04 | 59.51 | 82.96 | 36.3 |
| S50 | 110.14 | 76.1 | 133.39 | 38.6 |
| S51 | 191.82 | 326.89 | 338.42 | 41.7 |
| S52 | 232.77 | 200.97 | 254.39 | 35.1 |
| S53 | 348.81 | 265.06 | 292.38 | 32.5 |
| S54 | 130.83 | <50 | <50 | 40.6 |
| S55 | 161.2 | 59.7 | 83.5 | 34.6 |
| S56 | 281.18 | 108.78 | 200.89 | 38.7 |
| S57 | 145.31 | 73.62 | 125.73 | 33.6 |
| S58 | 105.1 | 69.71 | 113.48 | 33.3 |
| S59 | 164.48 | 67 | 105.03 | 42.5 |
| S60 | 187.87 | 77.89 | 138.77 | 31.4 |
| S61 | 151.8 | 90.12 | 170.35 | 36.4 |
| S62 | 225.46 | 89.93 | 160.95 | 38.2 |
| S63 | 119.01 | 59.37 | 82.57 | 34.7 |
| S64 | 86.8 | 67.22 | 105.72 | 36.9 |
| S65 | 87.75 | 70.17 | 114.91 | 32 |
| S66 | 211.52 | 139.45 | 226.57 | 33.6 |
| S67 | 235.32 | 103.52 | 194.03 | 40.1 |
| S68 | 125 | 55.79 | 72.52 | 39.8 |
| S69 | 256.36 | 88.4 | 166.52 | 33.5 |
| S70 | 120.76 | 63.44 | 94.32 | 36.1 |
| S71 | 236.47 | 257.48 | 287.25 | 33.4 |
| S72 | 101.94 | 113.17 | 205.85 | 38.3 |
| S73 | 90.36 | 60.63 | 86.16 | 36.5 |
| S74 | 86.26 | 60.83 | 86.73 | 39.2 |
| S75 | 108.69 | 64.62 | 97.21 | 35.8 |
| S76 | 98.06 | 70.34 | 115.45 | 41.4 |
| S77 | 122.84 | 52.88 | 64.52 | 35.4 |
| S78 | 224.39 | 86.72 | 162.59 | 32.9 |
| S79 | 144.14 | 69.85 | 113.9 | 40.2 |
| S80 | 55.52 | <50 | 52.93 | 34.3 |

Values reported as <50 U/mL were below the lower reportable limit and are presented as originally reported. The primary method-comparison analysis excluded the four paired observations containing at least one value reported as <50 U/mL, resulting in 76 fully quantifiable pairs. In the sensitivity analysis, values reported as <50 U/mL were replaced with 49.9 U/mL, allowing all 80 pairs to be included. Abbreviations: HCT, haematocrit; KL-6, Krebs von den Lungen-6.

**Supplementary Table 2.** Method-comparison analyses of venous and capillary plasma KL-6 measurements before and after HCT correction

| **Analysis** | **Comparison** | **n** | **Passing–Bablok intercept, U/mL (95% CI)** | **Passing–Bablok slope (95% CI)** | **Pearson r (95% CI)** | **Mean bias, U/mL** | **95% limits of agreement, U/mL** | **Wilcoxon signed-rank p value** |
| --- | --- | --- | --- | --- | --- | --- | --- | --- |
| Primary analysis | Uncorrected capillary plasma versus venous plasma | 76 | −13.59 (−37.10 to 10.65) | 0.769 (0.550–0.945) | 0.958 (0.934–0.973) | −48.32 | −164.78 to 68.15 | <0.001 |
| Primary analysis | HCT-corrected capillary plasma versus venous plasma | 76 | −7.03 (−26.97 to 9.76) | 1.004 (0.882–1.162) | 0.966 (0.947–0.978) | −3.47 | −104.42 to 97.49 | 0.149 |
| Sensitivity analysis | Uncorrected capillary plasma versus venous plasma | 80 | −8.01 (−26.49 to 16.15) | 0.726 (0.513–0.895) | 0.957 (0.933–0.972) | −47.08 | −162.02 to 67.87 | <0.001 |
| Sensitivity analysis | HCT-corrected capillary plasma versus venous plasma | 80 | −6.07 (−24.55 to 8.82) | 0.998 (0.885–1.152) | 0.966 (0.947–0.978) | −4.23 | −104.24 to 95.78 | 0.116 |

Notes: Venous plasma KL-6 was treated as the reference measurement and capillary plasma KL-6 as the comparison measurement. Bias was calculated as the capillary plasma measurement minus the venous plasma measurement. The 95% limits of agreement were calculated as the mean bias ± 1.96 SD of the paired differences. The primary analysis excluded four paired observations containing at least one result reported as <50 U/mL. In the sensitivity analysis, values reported as <50 U/mL were replaced with 49.9 U/mL, allowing all 80 paired observations to be included. Paired differences were assessed using the two-sided Wilcoxon signed-rank test. All Pearson correlation tests yielded p < 0.001. Abbreviations: CI, confidence interval; HCT, haematocrit; KL-6, Krebs von den Lungen-6; SD, standard deviation.

**Supplementary Table 3.** Agreement between HCT-corrected capillary and venous plasma KL-6 measurements across observed haematocrit strata

| **Observed HCT stratum** | **n** | **Observed HCT range, %** | **Mean bias, U/mL (95% CI)** | **Median absolute difference, U/mL (IQR)** |
| --- | --- | --- | --- | --- |
| ≤35.0% | 18 | 18.7–35.0 | −12.19 (−35.36 to 10.99) | 36.68 (16.18–56.23) |
| 35.1%–40.0% | 41 | 35.1–40.0 | −6.08 (−19.68 to 7.53) | 23.12 (13.54–52.48) |
| >40.0% | 17 | 40.1–44.1 | 12.05 (−24.76 to 48.86) | 33.70 (11.35–56.75) |

Notes: Bias was calculated as HCT-corrected capillary plasma KL-6 minus venous plasma KL-6. Absolute difference was defined as the absolute value of the paired corrected capillary-minus-venous difference. Corrected measurement differences did not differ significantly across the three observed HCT strata (Kruskal–Wallis p = 0.750). Across all 76 fully quantifiable pairs, HCT was not significantly correlated with corrected measurement difference (Spearman ρ = 0.115, p = 0.324) or absolute corrected difference (Spearman ρ = −0.017, p = 0.887). The observed HCT range was 18.7%–44.1%; therefore, these analyses do not establish performance at HCT values outside the range represented in this cohort. Abbreviations: CI, confidence interval; HCT, haematocrit; IQR, interquartile range; KL-6, Krebs von den Lungen-6.
